# A fast and robust iterative algorithm for prediction of RNA pseudoknotted secondary structures

**DOI:** 10.1186/1471-2105-15-147

**Published:** 2014-05-18

**Authors:** Hosna Jabbari, Anne Condon

**Affiliations:** 1 Department of Computer Science, University of British Columbia, 2366 Main Mall, Vancouver, Canada

**Keywords:** RNA, Secondary structure prediction, Pseudoknot, Hierarchical folding, Minimum free energy

## Abstract

**Background:**

Improving accuracy and efficiency of computational methods that predict pseudoknotted RNA secondary structures is an ongoing challenge. Existing methods based on free energy minimization tend to be very slow and are limited in the types of pseudoknots that they can predict. Incorporating known structural information can improve prediction accuracy; however, there are not many methods for prediction of pseudoknotted structures that can incorporate structural information as input. There is even less understanding of the relative robustness of these methods with respect to partial information.

**Results:**

We present a new method, Iterative HFold, for pseudoknotted RNA secondary structure prediction. Iterative HFold takes as input a pseudoknot-free structure, and produces a possibly pseudoknotted structure whose energy is at least as low as that of any (density-2) pseudoknotted structure containing the input structure. Iterative HFold leverages strengths of earlier methods, namely the fast running time of HFold, a method that is based on the hierarchical folding hypothesis, and the energy parameters of HotKnots V2.0.

Our experimental evaluation on a large data set shows that Iterative HFold is robust with respect to partial information, with average accuracy on pseudoknotted structures steadily increasing from roughly 54% to 79% as the user provides up to 40% of the input structure.

Iterative HFold is much faster than HotKnots V2.0, while having comparable accuracy. Iterative HFold also has significantly better accuracy than IPknot on our HK-PK and IP-pk168 data sets.

**Conclusions:**

Iterative HFold is a robust method for prediction of pseudoknotted RNA secondary structures, whose accuracy with more than 5% information about true pseudoknot-free structures is better than that of IPknot, and with about 35% information about true pseudoknot-free structures compares well with that of HotKnots V2.0 while being significantly faster. Iterative HFold and all data used in this work are freely available at http://www.cs.ubc.ca/~hjabbari/software.php.

## Background

RNA molecules are crucial in different levels of cellular function, ranging from translation and regulation of genes to coding for proteins [1-6]. Understanding the structure of an RNA molecule is important in inferring its function [7-10]. Since experimental methods for determining RNA structure, such as X-ray, crystallography and NMR, are time consuming, expensive and in some cases infeasible, computational methods for prediction of RNA structure are valuable.

Currently computational RNA structure prediction methods mainly focus on predicting RNA secondary structure—the set of base pairs that form when RNA molecules fold. When multiple homologous (evolutionarily related) RNA sequences are available, the secondary structure of the sequences can be predicted using multiple sequence alignment and comparative sequence analysis [11-22]. Alternative approaches, which can be used to predict secondary structure of a single sequence, are based on thermodynamic parameters derived in part from experimental data [23]. While thermodynamics-based approaches can be less accurate than comparative-based algorithms, thermodynamics-based approaches are applicable in cases of novel RNAs such as the many RNAs of unknown function recently reported by the ENCODE consortium [24]. Thermodynamics-based approaches can also be easier to apply to prediction of the structure of interacting RNA molecules, for example, in gene knockdown studies.

Many computational thermodynamics-based methods find the structures with the minimum free energy (MFE) from the set of all possible structures, when each structure feature is assigned a free energy value and the energy of a structure is calculated as the sum of the features’ energies. There has been significant success in prediction of *pseudoknot-free* secondary structures (structures with no crossing base pairs) [23,25,26]. While many small RNA secondary structures are pseudoknot-free, many biologically important RNA molecules, both in the cell [27,28], and in viral RNA [29] are found to be pseudoknotted.

Since finding the MFE pseudoknotted secondary structure is NP-hard [30-32], polynomial time MFE-based methods for prediction of pseudoknotted secondary structures predict a restricted class of pseudoknotted structures [33-35]. These methods trade off run-time complexity and the generality of the class of structures they can predict. For example, the most general algorithm of Rivas and Eddy [33], whose running time is *Θ*(*n*^6^) on inputs of length *n*, is not practical for RNA sequences of length more than 100 nucleotides. This has been the main reason for development of heuristic methods for prediction of pseudoknotted structures [36-41]. Although heuristic methods may not find the MFE structure, they usually run faster than the MFE-based methods that handle the same class of structures. For example, HotKnots V2.0 [36,41] is a heuristic approach that uses carefully trained energy parameters, is guided by energy minimization and can handle kissing hairpin structures. However, HotKnots is still slow on long sequences.

Other methods for prediction of pseudoknotted structures, such as the IPknot method of Sato et al. [42], are motivated by the finding of Mathews [43] that base pairs with high base pairing probabilities in the thermodynamic ensemble are more likely to be in the known structure. In a comprehensive comparison performed by Puton et al. [44] on the performance of publicly available non-comparative RNA secondary structure prediction methods that can handle pseudoknotted structures, IPknot ranks first for general length RNA sequences.

Incorporating known structural information can improve the accuracy of structure prediction. For example, Mathews et al. [45] used SHAPE reactivity data to improve the prediction accuracy from 26.3% to 86.8% for 5S rRNA of E. coli. Roughly, the larger the SHAPE reactivity value for a given nucleotide, the more likely it is that the nucleotide is unpaired in the structure. However, limited SHAPE reactivity data is available, and the data does not unambiguously determine whether a base is paired or not or, if it is paired, to what other nucleotide. Deigan et al. [46] created pseudo energy terms from SHAPE reactivity data, as a means of integrating such data into prediction software. They reported prediction accuracy of 96% to 100% for three moderate-sized RNAs (<200 nucleotides) and for 16S rRNA (1500 nucleotides). ShapeKnots [47] is a new method for incorporating SHAPE reactivity data for pseudoknotted structures that incorporates the pseudo energy terms into a heuristic method similar to that of Ren et al. [41].

We previously presented HFold [48], an approach for prediction of pseudoknotted structures, motivated by two goals, namely to avoid the high running time complexity of other methods for pseudoknotted secondary structure prediction and to leverage the *hierarchical folding hypothesis*. This hypothesis posits that an RNA molecule first folds into a pseudoknot-free structure; then additional base pairs are added that may form pseudoknots with the first structure so as to lower the structure’s free energy [49]. Given a pseudoknot-free structure as input, HFold predicts a possibly pseudoknotted structure from a broad class that contains the given input structure and, relative to that constraint, has minimum free energy. HFold’s running time is *O*(*n*^3^), significantly faster than other methods for predicting pseudoknotted structures. Several experts have provided evidence for, and support, the hierarchical folding hypothesis [49-52]. The class of structures that HFold can handle, density-2 structures, is quite general and includes many important pseudoknots including H-type pseudoknots, kissing hairpins and infinite chains of interleaved bands, with arbitrary nested (pseudoknotted) substructures. (Roughly, a structure is density-2 if no base is enclosed by more than two overlapping pseudoknotted stems.)

Another advantage of HFold over heuristic methods such as HotKnots or ShapeKnots is that unlike these methods, HFold minimizes the free energy of the possibly pseudoknotted output structure relative to the given input structure. Therefore HFold’s method of adding pseudoknotted stems is better motivated energetically than that of HotKnots or ShapeKnots.

While HFold is fast, our earlier implementation of HFold had its own shortcomings. First, due to a high pseudoknot initiation penalty in its underlying energy model, many of its predicted structures did not have pseudoknots. Also low band penalty (i.e., penalty for addition of pseudoknotted stems or bands) in its energy model encouraged addition of pseudoknotted stems when a pseudoknot was predicted. Second, if the first structure input to HFold contains base pairs that are not in the true pseudoknot-free structure for the given RNA sequence or is not the complete pseudoknot-free structure (i.e., it does not include all the base pairs in the pseudoknot-free structure), HFold is often unable to predict the known pseudoknotted structure as output.

To summarize, existing methods for prediction of pseudoknotted structures suffer from one or both of the following shortcomings: 1) slow running time, or 2) poor prediction accuracy. Moreover there is limited opportunity for the user to provide structural information, or constraints, that can guide prediction. In cases of a prediction method that incorporates user-defined constraints, it is also useful to understand the degree to which the method’s accuracy persists as the input information degrades. We use the term *robustness with respect to partial information* or *robustness* to refer to this property of a method. (We note that in our definition of robustness we do not mean robust with respect to noise.) To the best of our knowledge, the concept of robustness in secondary structure prediction methods has not been studied before.

In this work we present a new method that addresses these shortcomings. Our method, Iterative HFold, takes a pseudoknot-free input structure and produces a possibly pseudoknotted structure whose energy is at least as low as that of any (density-2) pseudoknotted structure containing the input structure. Iterative HFold incorporates four different methods and reports as its final structure the structure with the lowest energy, among all structures produced by these methods. While one of its underlying methods, HFold, strictly adheres to the hierarchical folding hypothesis, the other three use iterations to extend or remove the base pairs of input structure, with the goal of finding a structure that has lower energy than the structure found by HFold. Thus, unlike HFold, iterative HFold is able to modify the input structure (while the class of structures handled by both methods is the same). This is valuable since 1) computationally produced structures may not be completely accurate and 2) while the hierarchical folding hypothesis is a useful guiding principle, there is evidence that allowing for disruption of some base pairs in the initially formed pseudoknot-free secondary structure can improve prediction [53,54].

All of Iterative HFold’s underlying methods use the energy model of HotKnots V2.0 DP09 [36]; with this model, HFold obtained predictions with higher accuracy than those obtained with our earlier implementation of HFold. One of Iterative HFold’s underlying methods is HFold-PKonly, which given the input structure only adds pseudoknotted base pairs. HFold-PKonly is especially useful for cases when the user has either complete information about the true pseudoknot-free structure or wants to check whether a single stem of the input structure can be part of a pseudoknot since, if the input structure only has the specific stem in question, the output structure of HFold-PKonly will determine if the given stem can be part of a pseudoknot.

Based on our experiments on our HK-PK and HK-PK-free data sets that include 88 pseudoknotted structures, and 337 pseudoknot-free structures respectively, ranging in length from 10 to 400 nucleotides, a single run of Iterative HFold does not take more than 9 seconds time and 62 MB of memory. In contrast, one of the best heuristic methods, HotKnots V2.0, takes 1.7 hours and 91 GB of memory for a sequence with 400 nucleotides. Therefore our method is practical for prediction of long RNA structures. Iterative HFold bootstrap 95% percentile confidence interval for average accuracy of pseudoknotted structures of the HK-PK data set is significantly higher than that of IPknot, ((72.83%, 83.37%) vs. (54.56%, 66.25%)) and is comparable to that of HotKnots V2.0, (vs. (73.60%, 83.35%)) two of the best prediction methods available. Iterative HFold’s accuracy is significantly higher than that of IPknot and HotKnots on our IP-pk168 data set. Iterative HFold also has higher accuracy than HFold even when just partial information about the true pseudoknot-free structure is provided, so it is more robust than HFold. Specifically, Iterative HFold’s average accuracy on pseudoknotted structures steadily increases from roughly 54% to 79% as the user provides up to 40% of the input structure, and improves with a more modest but still positive improvement in accuracy when further structural information is provided.

## Methods

We represent an RNA molecule by a sequence, *S*, of its four bases, Adenine (A), Cytosine (C), Guanine (G) and Uracil (U). We denote the length of the RNA molecule by *n* and refer to each base by its index *i*, 1≤*i*≤*n*.

**Figure 1:**
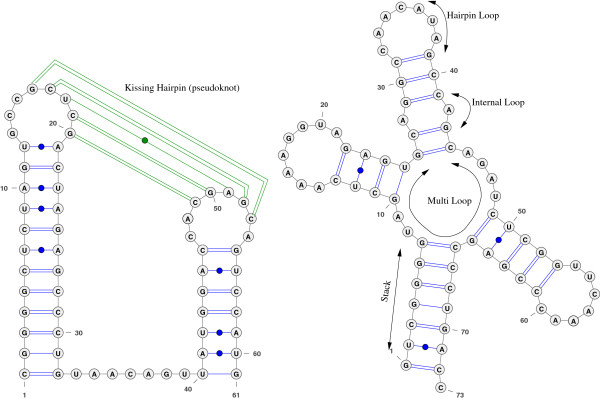
**Pseudoknotted and pseudoknot-free secondary structures.** Examples of loops and canonical base pairs in a pseudoknotted and a pseudoknot-free secondary structure. The blue base pairs belong to the *G*_*big*_ structure and the green base pairs belong to the *G*_*small*_ structure, as defined in Section ‘Definition of *G*_*big*_ and *G*_*small*_’. This figure was produced using the VARNA software [55].

When an RNA molecule folds, bonds may form between canonical pairs of bases (*A*-*U*, *C*-*G*, and *G*-*U*) (see Figure 1). Throughout this work, we consider only cases where each base may pair at most with one other base, and represent base pairing between *i* and *j* by *i*.*j*. We define a *secondary structure*, *R*, as a set of pairs *i*.*j*, 1≤*i*<*j*≤*n*; *i*.*j* and *k*.*j* can belong to the same set if and only if *i*=*k*.

If *i*.*j* and *k*.*l* are two base pairs of a secondary structure, *R*, and 1≤*i*<*k*<*j*<*l*≤*n*, we say *i*.*j* crosses *k*.*l*. We refer to a secondary structure with crossing base pairs as a *pseudoknotted secondary structure* and a secondary structure with no crossing base pairs as a *pseudoknot-free secondary structure* (see Figure 1). Figure 1 shows different kinds of loops in a secondary structure. We refer the readers to Jabbari et al. [48] or Rastegari et al. [56] for precise definition and illustration of terms used in the figure.

### Energy model

Many computational methods for predicting the secondary structure of an RNA (or DNA) molecule are based on models of the free energy of loops [23,25,26,33-36,48]. Table 1 summarizes the energy constants and functions used in our energy model for pseudoknotted structures. The values of these energy parameters are those of the DP09 parameter set of Andronescu et al. [36], used by the HotKnots V2.0 prediction software.

**Table 1:** Energy parameters

**Name**	**Description**	**Value (*Kcal/mol*)**
*P*_*s*_	Exterior pseudoloop	−1.38
	initiation penalty	
*P*_*sm*_	Penalty for introducing pseudoknot	10.07
	inside a multiloop	
*P*_*sp*_	Penalty for introducing pseudoknot	15.00
	inside a pseudoloop	
*P*_*b*_	Band penalty	2.46
*P*_*up*_	Penalty for unpaired base	0.06
	in a pseudoloop	
*P*_*ps*_	Penalty for closed subregion	0.96
	inside a pseudoloop	
*e*_*H*_(*i*,*j*)	Energy of a hairpin loop closed by *i*.*j*	
*e*_*S*_(*i*,*j*)	Energy of stacked pair closed by *i*.*j*	
*e*_*stP*_(*i*,*j*)	Energy of stacked pair that	0.89×*e*_*S*_(*i*,*j*)
	spans a band	
*e*_*int*_(*i*,*r*,*r*^′^,*j*)	Energy of a pseudoknot-free	
	internal loop	
*e*_*intP*_(*i*,*r*,*r*^′^,*j*)	Energy of internal loop	0.74×*e*_*int*_(*i*,*r*,*r*^′^,*j*)
	that spans a band	
*a*	Multiloop initiation penalty	3.39
*b*	Multiloop base pair penalty	0.03
*c*	Penalty for unpaired base	0.02
	in a multiloop	
*a*^′^	Penalty for introducing a multiloop	3.41
	that spans a band	
*b*^′^	Base pair penalty for a multiloop	0.56
	that spans a band	
*c*^′^	Penalty for unpaired base in a multiloop	0.12
	that spans a band	

This table provides the names, description and values of the energy parameters and functions that we used in our methods. The names and definitions are the same as in our original HFold [48], and the values were updated based on the work of Andronescu et al. [36]. These parameters were derived for a temperature of 37°C and 1 M salt concentration.

### Data sets

We use three data sets to analyze performance of our algorithms. Our first data set is the *test* data set of Andronescu et al. [36], that contains 446 distinct RNA sequences and their reference structures, of which 348 are pseudoknot-free and 98 are pseudoknotted. This set has four structures that are not in the class of structures our methods can handle (i.e., have densities higher than 2 [48]). Since the number of such structures is too small to be useful in an experimental analysis, we removed them from our set of pseudoknotted structures, resulting in a set of size 442.

There are eight cases in this data set for which the original sequence and structure were shortened to accommodate restrictions in length. We removed them from our data set, resulting in a set of size 425. From now on we use “HK-PK” to refer to the pseudoknotted structures in this set (with 88 structures) and “HK-PK-free” to refer to the pseudoknot-free structures in this set (with 337 structures). RNA sequences in HK-PK and HK-PK-free have length between 10 and 400 nucleotides.

Our second data set is the *pk168* data set of Sato et al. [42]. This set contains 168 pseudoknotted structures from 16 categories of pseudoknots. The sequences in this set have at most 85*%* similarity and have length of at most 140 nucleotides. We refer to this data set as “IP-pk168”.

Our third data set is the *test* data set of Sperschneider et al. [57]. This set contains 16 pseudoknotted structures with strong experimental support. RNA sequences in this set have length between 34 and 363 nucleotides. We refer to this data set as “DK-pk16”.

### Definition of *G*_*big*_ and *G*_*small*_

To test the robustness of our methods on a given RNA sequence, we need to provide partial information about the true pseudoknot-free structure as input structure for that sequence. To obtain the true pseudoknot-free structure, *G*_*big*_, we remove the minimum number of pseudoknotted base pairs from the reference structure to make the reference structure pseudoknot-free. If the reference structure is pseudoknot-free, then *G*_*big*_ is the same as the reference structure itself. We call the removed base pairs from the reference structure *G*_*small*_. Blue base pairs in Figure 1 represent base pairs of the *G*_*big*_ structure and green base pairs represent the *G*_*small*_ structure.

### Accuracy measures

Following common practice [36,58], we measure the accuracy of a predicted RNA secondary structure relative to a reference secondary structure by *F*-measure, which is the harmonic mean of *sensitivity* and *positive predictive value* (*PPV*). We define these values as follows:

$$\;\text{Sensitivity}=\frac{\mathrm{Number}\;\text{of}\;\text{correctly}\;\text{predicted}\;\text{base}\;\text{pairs}}{\mathrm{Number}\;\text{of}\;\text{base}\;\text{pairs}\;\text{in}\;\text{the}\;\text{reference}\;\text{structure}}$$

$$\text{PPV}=\frac{\mathrm{Number}\;\text{of}\;\text{correctly}\;\text{predicted}\;\text{base}\;\text{pairs}}{\mathrm{Number}\;\text{of}\;\text{predicted}\;\text{base}\;\text{pairs}}$$

and

$$F-\text{measure}=\frac{2\times \text{sensitivity}\times \text{PPV}}{\text{sensitivity}+\text{PPV}}$$

We also define these values as 0 when their denominators are 0. When a prediction agrees with the reference structure, the value of *F*-measure is equal to 1 (so are the values of sensitivity and PPV). When the values of sensitivity or PPV is equal to 0, the predicted structure does not have any base pairs in common with the reference structure.

### Bootstrap percentile confidence intervals

To formally assess the dependency of measured prediction accuracy of results of a method on a given set of RNA we use bootstrap confidence intervals, a well-known statistical resampling technique [59,60]. Following the recent work of Aghaeepour and Hoos [61] and Hajiaghayi et al. [58] we calculate the bootstrap 95% percentile confidence interval of average *F*-measure as follows. For each vector *f* of *F*-measures (where, for example, *f* may be the F-measures of predictions obtained by Iterative HFold on pseudoknotted structures) we first take 10^4^ resamples with replacement, where the resamples have the same length as the original sample vector *f* (|*f*|), and then calculate their average *F*-measures. These 10^4^ calculated average *F*-measures represent the bootstrap distribution for the vector *f*. We then report the 2.5th and 97.5th percentile of this distribution (i.e., the bootstrap distribution of the 10^4^ average *F*-measures calculated above) as the lower and upper bounds of the confidence interval respectively, and call it the bootstrap 95% percentile confidence interval. By reporting the bootstrap 95% percentile confidence interval for average *F*-measure of a method, *A*, on a data set, *D*, we say that we are 95% confident that the average *F*-measure of method *A* on data set *D* is in the reported interval. All calculations are performed using the “boot” package of the R statistics software environment [62].

### Permutation test

Following the recent work of Hajiaghayi et al. [58], we use a two sided permutation test to assess the statistical significance of the observed performance differences between two methods. The test proceeds as follows, given a data set and two structure prediction procedures, *A* and *B*. First, we calculate the difference *mean*(*f*_*A*_)−*mean*(*f*_*B*_) in means between sets of *F*-measure values obtained by *A* and *B*. Then we combine the two sets *f*_*A*_ and *f*_*B*_ and record the difference in sample means for 10^4^ randomly chosen ways of choosing two sets with the same size as |*f*_*A*_| and |*f*_*B*_| from the combined set. The *p*-value is the proportion of the sampled permutations where the absolute difference was greater than or equal to that of absolute difference of the means of sets *f*_*A*_ and *f*_*B*_. Then, if the *p*-value of this test is less than the 5% significance level, we reject the null hypothesis that methods *A* and *B* have equal accuracy and thus accept the alternative hypothesis that the difference in accuracy of method *A* and *B* is significant. Otherwise, we cannot reject the null hypothesis. All calculations are performed using the “perm” package of the R statistics software environment.

### Iterative HFold

We provide a high level description of our Iterative HFold algorithm.

Pseudocode of our Iterative HFold algorithm is available in Additional file 1. The algorithm builds on two simpler methods, the first being our original HFold algorithm [48]: ***HFold:*** Given an RNA sequence, *S*, and a pseudoknot-free input structure, *G*, find a pseudoknot-free structure, *G*^′^ such that *G*∪*G*^′^ is the lowest energy structure that contains *G*. We note that *G*∪*G*^′^ might not be pseudoknotted.

The second method on which Iterative HFold builds, called HFold-PKonly, is similar to HFold except that *G*^′^ may only contain base pairs that cross base pairs in *G*. The prediction provided by HFold-PKonly can be useful in cases where HFold does not produce a pseudoknotted structure. ***HFold-PKonly:*** Given an RNA sequence, *S*, and a pseudoknot-free input structure, *G*, find a pseudoknot-free structure, *G*^′^ such that every base pair in *G*^′^ crosses some base pair of *G* and such that *G*∪*G*^′^ is the lowest energy structure that contains *G* among all such *G*^′^s. Note that *G*^′^ may contain no base pairs.

Iterative HFold also uses the SimFold RNA secondary structure prediction method [63], which predicts the minimum free energy pseudoknot-free secondary structure for a given RNA sequence. SimFold uses a dynamic programming method similar to Zuker’s MFold method [64]. In this work we used the HotKnots energy parameters when running SimFold. In addition to an RNA sequence, *S*, SimFold can also take a pseudoknot-free secondary structure, *G*, as input and predict the MFE pseudoknot-free secondary structure that contains all base pairs of *G*.

Iterative HFold is distinguished from the above three methods, namely HFold, HFold-PKonly and SimFold, in two important ways. First, the output of HFold, HFoldPKonly and SimFold methods must contain the given pseudoknot-free input structure, *G*, whereas Iterative HFold may modify the input structure. This can be useful when the given input structure is not a high-accuracy estimate of *G*_*big*_, the true pseudoknot-free substructure of the reference structure. Second, while HFold and HFold-PKonly can add base pairs that cross those in *G*, they cannot add base pairs that cross each other, and neither can SimFold. In contrast, Iterative HFold can add base pairs that cross each other. This is particularly useful when the input structure contains limited information about *G*_*big*_, and so it is necessary both to predict base pairs in *G*_*big*_ and in *G*_*small*_ in order to get a good prediction.

Iterative HFold is comprised of four different iterative methods. Following the description of each method, we motivate why we chose to include it as part of our overall algorithm. Iterative HFold takes as input both an RNA sequence, *S* and a pseudoknot-free secondary structure, *G*; later we show that structure *G* can be produced by computational methods, for example, HotKnots hotspots or SimFold suboptimal structures, when only the sequence *S* is initially available. ***Iterative HFold:*** Given an RNA sequence, *S*, and a pseudoknot free input structure, *G*, run the following four methods and pick the structure with the lowest free energy among these four as the output structure.

Iterative HFold runs in *O*(*n*^3^) time, as it runs four methods sequentially, when each one is *O*(*n*^3^). 

*Method 1:* Run HFold on *S* and *G*, and store the resulting *G*∪*G*^′^.

*Motivation:* This is the core HFold method, motivated by the hierarchical folding hypothesis.

*Method 2:* First run HFold-PKonly on *S* and *G*. If HFold-PKonly results in a structure *G*∪*G*^′^ such that *G*^′^ is not the empty structure, then run HFold with sequence *S* and structure *G*^′^, and store the result. Otherwise, simply store *G* as the result. See the following example. (We note that running HFold with *S* and *G*^′^ results in a structure *G*^′^∪*G*^′′^, where it may be the case that *G*^′′^≠*G* (i.e., *G* may not be part of the result of method 2).)

*Motivation:* When input structure *G* does not agree with the reference *G*_*big*_ structure, it may still be the case that HFold-PKonly finds the pseudoknotted structure *G*_*small*_ (or a good approximation to *G*_*small*_). A call to HFold with input *G*_*small*_ may then find a better approximation to *G*_*big*_.

**Example 1**: Example of results of *method 1* and *method 2* of Iterative HFold. 

 In this example, method 2 of Iterative HFold outperforms method 1: although both HFold and HFold PKonly produce the same result on sequence *S* and input structure *G*, namely the structure $G\cup G^{{}^{\prime }}$, the additional iteration in method 2, in which HFold is run with *S* and $G^{{}^{\prime }}$, finds a structure with lower energy than that of $G\cup G^{{}^{\prime }}$.

*Method 3:* First run *SimFold* on *S* and *G* to obtain result *G*^′^—a pseudoknot-free structure that contains *G*. Then let *G*_*updated*_ be the secondary structure of *S* containing the relaxed stems of *G*^′^ that include the base pairs of *G*. By a *relaxed stem*, we mean a secondary structure containing stacked base pairs, bulges of size 1 and internal loops of maximum size of 3 (i.e., either the symmetric loop of 1×1 or the non-symmetric loop of 1×2 or 2×1 but no other loop types; this is motivated by common practice [65]). Then run *method 2* on *S* and *G*_*updated*_, and store the result. See Example 2.

*Motivation:* This method can work well when the given input structure has a small number of base pairs from *G*_*big*_, because *G*_*updated*_ contains stems that includes these base pairs, but avoids “overcrowding” with further base pairs that might prevent HFold-PKonly from finding pseudoknotted stems.

**Example 2:** Example of result of *method 3* compared to all four methods of Iterative HFold. 

 In this example, method 3 of Iterative HFold outperforms the other methods. Because the input structure *G* consists of just one base pair, method 1 (HFold) outputs a pseudoknot-free structure containing *G*. The output of both methods 2 and 4 are pseudoknotted but do not contain the base pair of the input structure *G*. In contrast, method 3 first adds base pairs to *G*, resulting in the pseudoknot-free structure *G*_*updated*_, and then adds additional pseudoknotted base pairs via method 2.

*Method 4:* Let *S*_1_ be the subsequence of *S* obtained by removing bases that are external unpaired bases with respect to input structure *G*. Run *SimFold* on *S*_1_ and *G* (with base indices renumbered to agree with *S*_1_), to obtain pseudoknot-free structure *G*^′^. Then continue exactly as in method 3. See Example 3.

*Motivation:* This method is very similar to method 3, but further constrains *G*^′^ since the base pairs in *G*^′^ cannot involve bases that are removed from *S* to obtain *S*_1_. This potentially increases the possibilities for pseudoknotted base pairs to be added by method 2.

**Example 3:** Example of result of *method 4* compared to all four methods of Iterative HFold. 

 In this example, method 4 of Iterative HFold outperforms the other methods. The input structure *G* has a high energy value and neither method 1 (HFold) nor method 2 (HFold-PKonly) can expand the pseudoknot-free structure to add the pseudoknotted stem. Also, by adding too many pseudoknot-free base pairs, method 3 fails to find the pseudoknotted base pairs. Thus, method 4 performs better than methods 1, 2 and 3.

### Experimental settings

In this section we explain details of our computational experiments.

#### Robustness test

One of our goals is to understand the degree to which our methods are *robust with respect to partial information*, that is, provide a reliable prediction even when limited information about the true pseudoknot-free structure, *G*_*big*_, is available. For this purpose we generate subset structures of the corresponding *G*_*big*_, for each RNA sequence in the HK-PK and HK-PK-free data sets. For each *α*, 0.05≤*α*≤0.95 with 0.05 steps, we choose each base pair of *G*_*big*_ structure with probability *α*. We also generate 1*%* information and 99*%* information about the *G*_*big*_ structure (i.e., *α*=0.01 and *α*=0.99). We repeat this step 100 times to generate 100 substructures of *G*_*big*_ for each value of *α* for each RNA sequence in our data sets. We then run our methods on all 100 substructures for each RNA sequence in our data sets and *α* value and calculate the bootstrap 95*%* percentile confidence interval for average F-measure of these 100 cases as the accuracy interval for each method and each RNA sequence and *α* value in our data set.

We also compare our methods when the true pseudoknot-free structure, *G*_*big*_ is provided.

#### Accuracy comparison tests

We compare the accuracy of HFold, HFold-PKonly and Iterative HFold with each other on different input structures, and with other methods, namely SimFold [63], HotKnots V2.0 [36,41] and IPknot [42]. We first describe the latter two methods and the settings we choose for our experiments. We then describe the ways in which we choose input structures for HFold and its variants.

##### HotKnots

HotKnots is a heuristic program that given an RNA sequence, first finds about 20 lowest energy stems (from the set of all stems for the given RNA sequence), called *hotspots*. Then keeping all these stems, it adds other non-overlapping low energy stems to the stems found in the first step, so as to minimize the energy of the overall structure, eventually producing up to 20 output structures. In our experiments, we choose the structure with the lowest energy value among the 20 output structures as the final structure predicted by HotKnots. When reporting prediction accuracy for HotKnots, we report the bootstrap 95*%* percentile confidence interval for the average F-measure of the lowest energy structure for all RNA sequences in our data set.

##### IPknot

IPknot is a secondary structure prediction method based on Maximum Expected Accuracy (MEA) of the base pairs. In addition to the RNA sequence, IPknot gets several parameters as input. Following, we describe each of these parameters and settings briefly. 

•level: If structure *G* can be decomposed into *k* disjoint pseudoknot-free structures, *G*_1_,*G*_2_,…,*G*_*k*_, such that every base pair in *G*_*i*_ crosses the base pairs of *G*_*j*_, 1≤*i*≤*j*≤*k*, Sato et al. say that structure *G* has *k* levels. For example, a pseudoknot-free structure has level 1, and an H-type pseudoknot has level 2. In another example, when representing the secondary structure in dot bracket format, the number of different brackets used to represent the structure is the level of the structure. IPknot can handle structures up to level 3.

•scoring model: The energy model used to produce posterior probabilities for each base pair is called “scoring model”. IPknot has 3 different scoring models, namely “CONTRAfold”, “McCaskill” and “NUPACK”.

•refining parameters: The procedure of recalculating the base pair probabilities based on the original prediction results is referred to as “refining parameters”.

•base pair weights for each level: Positive numbers representing the rate of true base pairs in each level.

We run IPknot using the provided source code and the default parameters for scoring model and level (i.e., scoring model = McCaskill and level =2). The default values provided for base pair weights are not the same on the IPknot website (i.e., *γ*_1_=2 and *γ*_2_=16), its source code (i.e., for some cases *γ*_1_=2 and *γ*_2_=4 and for others *γ*_1_=1 and *γ*_2_=1) and the provided perl script (i.e., *γ*_1_=4 and *γ*_2_=8). We run IPknot with all of these values with and without refinement and provide IPknot’s bootstrap 95*%* confidence intervals for average F-measures for all of our data sets as a table in the Additional file 2. Based on its performance we present IPknot’s results with default settings (i.e., no refinement, scoring model = McCaskill and level =2) and *γ*_1_=4 and *γ*_2_=8, for comparison with other methods.

##### Different versions of HFold

We compare the average accuracy of HFold, HFold-PKonly and Iterative HFold with different input structures.

To determine which input structures are good to use when *G*_*big*_ is not known, we compare two different options. Since HFold (HFold-PKonly and Iterative HFold) cannot accept pseudoknotted input structures we use the following methods to produce pseudoknot-free input structures to HFold (HFold-PKonly and Iterative HFold). First, we use HotKnots hotspots [36], i.e., the 20 lowest energy pseudoknot-free stems produced in the first phase of HotKnots. We choose the lowest free energy structure predicted by each of our methods as their final prediction given these hotspots. Second, we use SimFold’s MFE structure [63] where the energy parameters of SimFold are changed to match that of HotKnots V2.0.

#### Running time

We ran all methods on the same platform (Macbook pro. OS X 10.5.8 with 2.53 GHz Intel Core 2 Duo processor and 4 GB 1067 MHz DDR3 RAM). We use the *time* command to measure the running time of our methods on each sequence, and record the wall clock time.

#### Memory usage

To find the memory usage of the programs, we use the Valgrind package [66] and record the total heap usage as memory usage of each program. IPknot and HotKnots are completely written in C and so we can easily find their memory usage by running Valgrind. However, Iterative HFold program is a perl script that runs a few C programs (HFold, HFold-pkonly and SimFold) sequentially. So we find the memory usage of each C component using Valgrind and assign the maximum as the memory usage of Iterative HFold.

## Results

As mentioned in Section ‘Background’, in the literature on hierarchical folding, there are reports of counter examples to the hierarchical folding hypothesis where bases that are initially part of the pseudoknot-free structure for a molecule later change as the pseudoknot forms. This motivates a comparison of HFold versus Iterative HFold, in order to see how a method that sticks strictly with the hypothesis (i.e., HFold) compares with a method that allows for some base changes (i.e., Iterative HFold). In Section ‘Robustness comparison’, we compare the robustness of HFold and Iterative HFold with respect to partial information; that is, the degree to which they provide accurate predictions as a function of how much information about *G*_*big*_, the true pseudoknot-free secondary structure, is provided as input. Then in Section ‘Accuracy comparison of different versions of HFold’ we compare HFold, HFold-PKonly and Iterative HFold when a (possibly inaccurate) computational prediction of *G*_*big*_ is provided as input. In Section ‘Accuracy comparison with existing methods’ we compare Iterative HFold—the method that performs best overall in Sections ‘Robustness comparison’ and ‘Accuracy comparison of different versions of HFold’ —with existing methods for pseudoknotted secondary structure prediction. Sections ‘Running time comparison’ and ‘Memory consumption comparison’ report on the running time and memory usage of our methods.

### Robustness comparison

**Figure 2:**
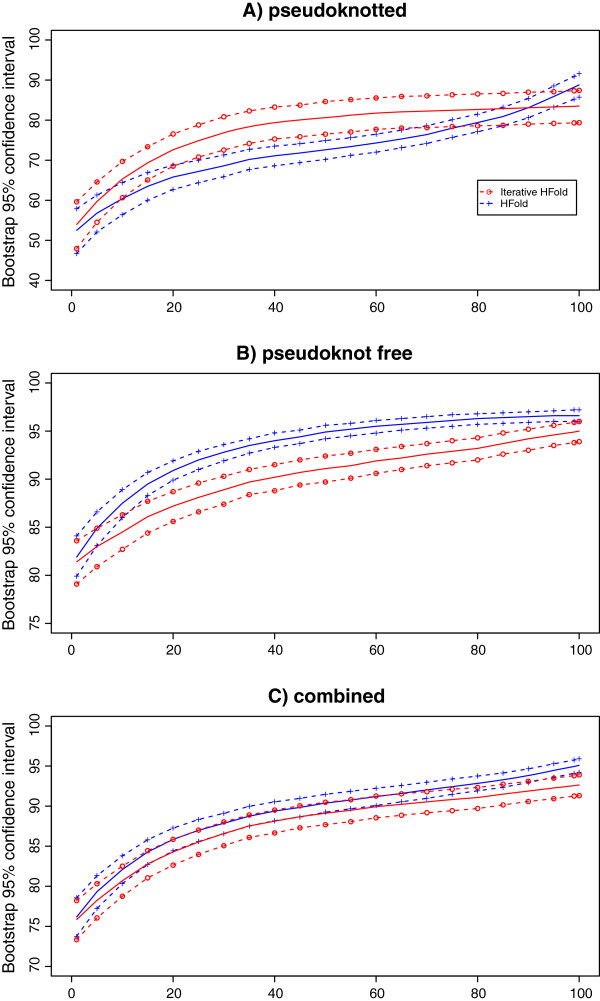
**Comparison of robustness of HFold and Iterative HFold.** Robustness results for pseudoknotted structures of the HK-PK data set **(**2**A)**, pseudoknot-free structures of the HK-PK-free data set **(**2**B)** and all structures **(**2**C)**. The X axes show the available information about *G*_*big*_ structure in percentage format, and the Y axes show bootstrap 95% percentile confidence intervals for average F-measure. Dashed lines show the borders of the bootstrap 95% percentile for average F-measure and solid lines show the average F-measure itself.

One of our goals is to learn what is the accuracy of each of our methods when partial information about *G*_*big*_ is available (see Section ‘Robustness test’ for experimental settings). Figure 2 shows the results of this robustness evaluation, for pseudoknotted structures (Figure 2A), pseudoknot-free structures (Figure 2B) and the overall results (Figure 2C). Since HFold-PKonly cannot add pseudoknot-free base pairs to the given input structure, we do not compare its performance here with HFold and Iterative HFold. However we provide detailed performance of all versions of HFold including HFold-PKonly in Additional file 3.

As shown in Figure 2A, which pertains to pseudoknotted structures of the HK-PK data set, when provided with ≈1*%* of the *G*_*big*_ structure as input, Iterative HFold’s bootstrap 95*%* percentile confidence interval of average F-measures has higher accuracy than those of HFold. Iterative HFold continues to be significantly superior to HFold until approximately 90*%* of *G*_*big*_ is available, after which HFold is more accurate. Iterative HFold is most successful when little information about *G*_*big*_ is known because it can add both pseudoknot-free and pseudoknotted base pairs. In particular, using methods 3 and 4 (see Section ‘Iterative HFold’) Iterative HFold first finds a low energy pseudoknot-free structure that includes the given input structure (by extending the stems of the given structure), and then adds pseudoknotted base pairs to further lower the energy of the overall structure. However, when the vast majority of base pairs of *G*_*big*_ are provided as input, HFold dominates as it keeps the base pairs of the input structure, thereby often adding base pairs of *G*_*small*_. When 100*%* of *G*_*big*_ is provided as input, HFold’s bootstrap 95*%* percentile confidence interval is (85.74*%*,91.87*%*), compared with (79.36*%*,87.41*%*) for Iterative HFold.As shown in Figure 2A, Iterative HFold’s average accuracy on pseudoknotted structures steadily increases from about 54% to 79% as the user provides 1% to 40% of the input structure. This improvement in accuracy slows down but still persists when further structural information is provided. If we compare the slope of the curve for Iterative HFold’s average accuracy to that of HFold in Figure 2A, we can see that HFold’s slope is steeper than that of Iterative HFold, making Iterative HFold more robust than HFold.

For pseudoknot-free structures of the HK-PK-free data set, as shown in Figure 2B, HFold performs better than Iterative HFold. Even with 1*%* information about *G*_*big*_, HFold results in (79.76*%*,84.32*%*)95*%* bootstrap confidence interval in comparison with (79.14*%*,83.84*%*) for Iterative HFold with the same inputs. Roughly, HFold’s success for pseudoknot-free structures is because it often adds base pairs that do not cross those provided as part of the input, and thus are likely to be in *G*_*big*_.

When 100% of *G*_*big*_ is provided as input, the overall bootstrap 95*%* confidence interval for HFold is (96.11*%*,97.24*%*) compared with (93.85*%*,96.07*%*) for Iterative HFold.

### Accuracy comparison of different versions of HFold

Often, partial information about *G*_*big*_ is not available; this is the case for many RNAs of unknown function reported by the ENCODE consortium [24]. Therefore, we next compare the quality of results obtained by HFold, HFold-PKonly and Iterative HFold when given a pseudoknot-free input, *G* that is predicted by existing computational methods. One way to produce an input structure is to use an MFE pseudoknot-free structure prediction method, such as MFold. We chose SimFold as it is an implementation of MFold and, because of its energy parameters, gives more accurate predictions than MFold. Of course, when comparative information is available, the user can input such information as structural constraint as a pseudoknot-free structure to Iterative HFold and expect a better prediction result. Here we compare two methods for predicting *G*, namely SimFold and the *hotspots* produced by HotKnots V2.0. Table 2 reports the bootstrap 95*%* percentile confidence intervals of average F-measures. The accuracy of HFold-PKonly is significantly worse than that of HFold and Iterative HFold, both with the output of SimFold, and with the HotKnots hotspots as input, so we do not discuss HFold-PKonly further.

**Table 2:** Comparison of bootstrap 95% percentile confidence interval of average F-measure of different versions of HFold when given SimFold structure as input vs. when given HotKnots hotspots structures as input

**Input**	**Hotspots**			**SimFold (MFE)**		
	**PKonly**	**HFold**	**Iter. HFold**	**PKonly**	**HFold**	**Iter. HFold**
HK-PK	(55.54, 71.06)	(73.35, 83.53)	(72.83, 83.37)	(50.57, 63.53)	(50.69, 63.54)	(51.42, 64.39)
HK-PK-free	(31.37, 38.52)	(75.53, 80.79)	(74.93, 80.26)	(78.42, 83.21)	(78.33, 83.27)	(78.31, 83.17)

For pseudoknotted structures, using HotKnots hotspots as input is far superior to using SimFold as input, for both HFold and Iterative HFold. This appears to be because MFE structures predicted by SimFold tend to have more base pairs than the true pseudoknot free structure, *G*_*big*_, so that HFold and Iterative HFold are unlikely to add pseudoknotted base pairs to the input structure. For pseudoknot-free structures, using SimFold as input is somewhat better than using HotKnots hotspots, but the permutation test indicates that the difference is not significant.

The confidence intervals for HFold and Iterative HFold with HotKnots hotspots are (73.35%, 83.53%) and (72.83%, 83.37%), respectively, and on pseudoknot-free structures they are (75.53%, 80.79%) and (74.93%, 80.26%) respectively. Again, based on the result of the permutation test, the difference in the results of HFold and Iterative HFold on pseudoknotted and pseudoknot-free structures are not significant. Similarly, the permutation test shows that the difference in prediction accuracy of HFold and Iterative HFold on SimFold input is not significant.

### Accuracy comparison with existing methods

For comparisons with other methods already in the literature, we choose to use our Iterative HFold method with HotKnots hotspots as input structure, based on its overall good accuracies in Section ‘Accuracy comparison of different versions of HFold’. We compare this method with two of the best-performing methods [44] for prediction of pseudoknotted structures, namely HotKnots V2.0 [36], a MFE-based heuristic method, and IPknot [42], a method that is based on maximum expected accuracy. (Prepared by Puton et al. [44], CompaRNA, is the website for continuous comparison of RNA secondary structure methods on both PDB data set and RNA strand. We chose IPknot because it was the best-performing non-comparative pseudoknot prediction method that can handle long RNA sequences, based on the ranking on their website as of March 25, 2014. We also noticed that Puton et al. used HotKnots V1 for their comparison, and not the more recently available and better performing HotKnots V2.0. Therefore we chose to include HotKnots in our comparisons as well. Since the focus of this paper is on prediction of pseudoknotted structures, we do not compare our results with that of Co-Fold [26] or other methods for prediction of pseudoknot free structures.)

Table 3 presents the bootstrap 95% percentile confidence interval of average F-measure for Iterative HFold with hotspots as input, HotKnots V2.0, SimFold and IPknot with default setting (see Section ‘Accuracy comparison tests’) on the HK-PK and HK-PK-free data sets. For pseudoknotted structures, our permutation tests show that the difference in accuracy of Iterative HFold and HotKnots is not significant. However, the superior accuracy of Iterative HFold compared with SimFold and IPknot is significant. For pseudoknot-free structures, the difference in accuracy between IPknot, Iterative HFold, HotKnots and SimFold is not significant.

**Table 3:** Comparison of bootstrap 95% percentile confidence interval of average F-measure with existing methods

**Input**	**Iter. HFold**	**HotKnots**	**SimFold**	**IPknot**
	**(hotspots)**			**(default)**
HK-PK	(72.83, 83.37)	(73.60, 83.35)	(45.34, 57.73)	(54.56, 66.25)
HK-PK-free	(74.93, 80.26)	(76.74, 81.95)	(78.78, 83.55)	(77.31, 81.79)

Table 4 presents the bootstrap 95*%* percentile confidence interval for average F-measure for Iterative HFold (with hotspots as input), HotKnots and IPknot (with default setting) on the IP-pk168 and DK-pk16 data sets. Our permutation tests show that the difference in accuracy of Iterative HFold, HotKnots and IPknot on the DK-pk16 data set is not significant. However, the superior accuracy of Iterative HFold compared with HotKnots and IPknot on the IP-pk168 data set is significant.

**Table 4:** Comparison of bootstrap 95% percentile confidence interval of average F-measure with existing methods on the DK-pk16 and the IP-pk168 data sets

**Input**	**Iter. HFold**	**HotKnots**	**IPknot**
	**(hotspots)**		**(default)**
DK-pk16	(68.05, 81.85)	(69.11, 83.81)	(65.42, 75.81)
IP-pk168	(72.65, 79.86)	(65.51, 72.96)	(58.20, 66.09)

### Running time comparison

**Figure 3:**
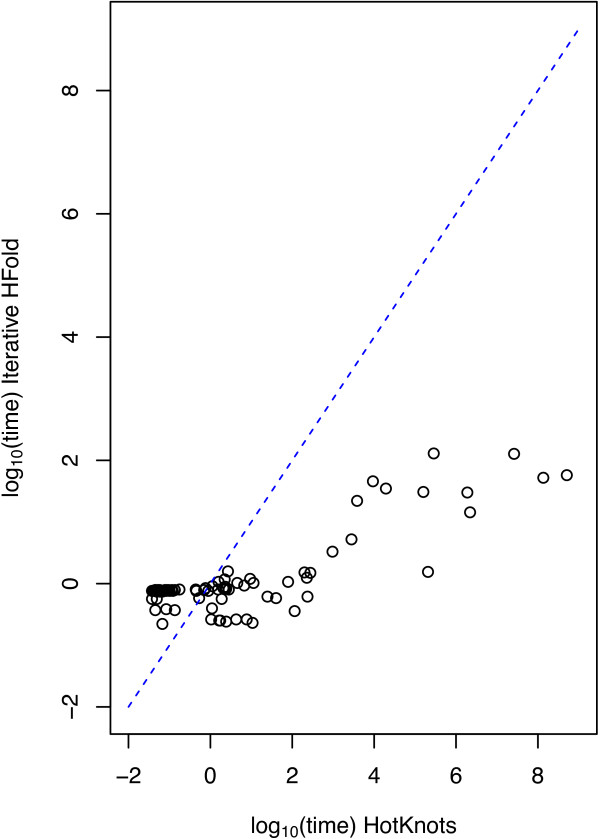
**Time comparison.** Comparison of running times of Iterative HFold and HotKnots in a log plot. The X axis shows log10(*time*) for HotKnots data points and the Y axis shows log10(*time*) for Iterative HFold. Time is measured in seconds.

Since prediction of pseudoknotted structures are of interest to us, we only report running time comparison on pseudoknotted structures of our HK-PK data set. Figure 3 presents result of time comparison between Iterative HFold and HotKnots in a log plot (when *log* is in base 10). The X axis shows log(*time*) for HotKnots data points and the Y axis shows log(*time*) for Iterative HFold on the HK-PK data set. RNA sequences in this data set are between 26 and 400 bases long. HFold runs significantly faster than HotKnots and finishes under 1.5 seconds for even the longest RNA sequence in our data set (400 bases). HotKnots is faster than Iterative HFold on sequences of up to 47 bases, where Iterative HFold starts being faster than HotKnots. Iterative HFold runs in less than 8.3 seconds for all RNA sequences in this data set whereas HotKnots runs for over 6000 seconds (about 1.7 hours) on the longest RNA sequence in our data set. The running time of both HFold and Iterative HFold grows with sequence length, whereas HotKnots’ running time is not directly correlated with RNA length. For example, HotKnots runs for 1665.94 seconds for one RNA sequence of length 195 (ASE-00360), while it runs for 203.12 seconds for another RNA sequence of length 195 (ASE-00131).

IPknot is significantly faster than both HFold and Iterative HFold. For all sequences in this data set, IPknot produces output in less than 0.8 seconds. For detailed information about performance of each method see Additional file 4.

### Memory consumption comparison

Here we present memory consumption of HFold, Iterative HFold and HotKnots on our HK-PK pseudoknotted structures. Since HotKnots predicts and keeps about 20 structures in memory, its memory consumption can vary significantly from one sequence to another, and is not predictable. Up until 47 bases, HotKnots some times uses less memory than HFold or Iterative HFold, but for RNA sequences with 47 bases or longer, HotKnots uses much more memory than HFold and Iterative HFold. Iterative HFold’s memory usage is very similar to HFold’s and increases at a very low rate by the length of the RNA sequence. It starts from 48.69 MB for RNA sequences of length 26 and increases to 61.33 MB for the longest RNA sequence in this data set (400 bases long). HotKnots, however, uses as little as 16.53 MB for an RNA of length 30 bases (LP-PK1) and as much as 93419 MB for the longest RNA sequence in this data set.

IPknot uses much less memory than all other methods. For the longest RNA sequence in this data set, IPknot uses less than 5.5 MB of memory in comparison to 61.33 MB of HFold and Iterative HFold and 93419 MB of HotKnots. For detailed information about memory usage of each method see Additional file 4.

## Discussion

In Section ‘Comparison with Hotknots and IPknot’ we provide more insight on the differences and merits of Iterative HFold, HotKnots and IPknots. Then in Section ‘Comparison with ShapeKnots’ we compare accuracy of Iterative HFold with ShapeKnots, a method that incorporates SHAPE reactivity data to predict RNA pseudoknotted secondary structure. In Section ‘Iterative HFold with SimFold’s suboptimal structures’ we compare performance accuracy of Iterative HFold with two inputs: HotKnots hotspots and suboptimal structures. Section ‘Energy model’ provides more insight into the energy model used in this work.

### Comparison with Hotknots and IPknot

Comparing accuracy of Iterative HFold and HotKnots V2.0 on HK-PK, HK-PK-free, DK-pk16 and IP-pk168, we found that the difference in their accuracies is insignificant on HK-PK, HK-PK-free and DK-pk16 data sets when Iterative HFold is provided with HotKnots hotspots as input. Based on our results on the HK-PK data set, with only about 15*%* information about the true pseudoknot-free structures, Iterative HFold’s 95% percentile confidence interval is (65.08*%*;73.36*%*) (data shown in Additional file 3). If the user has about 35% information about the true pseudoknot-free structure, Iterative HFold’s accuracy is comparable with that of HotKnots (i.e., (74.18*%*;82.30*%*) vs. (73.60*%*;83.35*%*)). However Iterative HFold’s accuracy (with hotspots as input) is significantly better than that of HotKnots on the IP-pk168 data set. One of the advantages of Iterative HFold over HotKnots is that in Iterative HFold base pairs are added to lower the energy of the given structure while in HotKnots stems are added in a way that does not take into account the energy of stems in the previous steps.

When reporting on time and memory consumption of Iterative HFold and HotKnots on the HK-PK data set, we did not include the time and memory required to get the input structures to Iterative HFold. Since we only run HotKnots V2.0 partially to produce hotspots it does not take as long as running HotKnots and does not consume as much memory. For example, for the 400 nucleotides long RNA sequence in our data set (A.tum.RNaseP), it only takes 0.5 seconds time and 4 MB of memory to produce the hotspots. (The time required to get the hotspots for all RNA sequences in this data set is provided in Additional file 4.) We also note that since calculating hotspots and running Iterative HFold are done sequentially, the memory consumption is calculated as the maximum of the two, so the memory consumption of Iterative HFold for this sequence is still the same even including the memory needed for calculating hotspots. As we can see based on this example, even including time and memory requirements of calculating hotspots, Iterative HFold is still faster than HotKnots and uses less memory.

We also compared Iterative HFold with IPknot [42]. While IPknot is faster than Iterative HFold and uses less memory, we found that for the HK-PK and IP-pk168 data sets, Iterative HFold provides significantly more accurate predictions of pseudoknotted structures, compared with IPknot. Based on our results on the HK-PK data set, Iterative HFold’s performance with more than 5% information about the true pseudoknot-free structure, is better than that of IPknot with default settings (data shown in Additional file 3). We note that Sato et al. [42] find the performance of IPknot with predictions using “NUPACK” superior to all versions of IPknot, but since this model can be used for RNA sequences of length <80 nucleotides, we did not compare our results with this version of IPknot. Among all different versions of IPknot we tested, we found all but *γ*_1_=1 and *γ*_2_=1 setting producing similar confidence intervals for all but the HK-PK-free data sets, for which *γ*_1_=4 and *γ*_2_=8 produces the best result (data shown in Additional file 2). While running parameter refinement with one iteration improved the confidence intervals in the HK-PK data set, it did not result in any improvement in accuracy in the rest of our data sets as in many cases IPknot failed to produce results. We note that our results on the IP-pk168 data set with different weight parameters perfectly match the results of Sato et al. [42].

A disadvantage of IPknot over Iterative HFold is that being an MEA-based method, IPknot does not produce free energy of the predicted structure. Also to get the best prediction, the user needs to provide some guidance as to what type of structure to predict for the given sequence, e.g., whether pseudoknot-free or pseudoknotted.

### Comparison with ShapeKnots

Similar to HotKnots, the ShapeKnots method of Hajdin et al. [47] is a heuristic algorithm for prediction of pseudoknotted structures. This method incorporates SHAPE reactivity data as a pseudo energy term into the prediction method. SHAPE reactivity data is only available for a limited number of RNA sequences, so we cannot compare Iterative HFold with ShapeKnots on our data set. Therefore, we use data set of Hajdin et al. to compare these two methods. In their data set Hajdin et al. have 18 RNA sequences in their training set and 6 RNA sequences in their test set. We run Iterative HFold with hotspots for each RNA sequence and choose the lowest energy structure as the final output of our program. For Shapeknots, we use the sensitivity and positive predictive values reported in the work of Hajdin et al. [47] to compare with Iterative HFold. Table 5 shows the results of this comparison. In all but one sequence of the test set, Iterative HFold obtains higher accuracy than ShapeKnots. The exception is the HIV-1 5’ pseudoknot domain; Hajdin et al. note that the accepted structure of HIV-1 5’ pseudoknot domain is based on a SHAPE directed prediction and thus an accuracy comparison between ShapeKnots and Iterative HFold may be biased towards ShapeKnots. In the training set, however, Iterative HFold does not perform as well as ShapeKnots. This might be because parameters of ShapeKnots were tuned on the training set to achieve the highest possible accuracy. Since both the training and test data sets are small, we cannot make more general statements about the significance of the differences in accuracy between the two methods.

**Table 5:** Comparison of Iterative HFold F-measure with ShapeKnots on SHAPE data

**Training set**	**Len**	**PK**	**Iter. HFold**			**ShapeKnots**		
			**sen**	**ppv**	**F**	**sen**	**ppv**	**F**
Pre-Q1 riboswitch, B. subtilis	34	1	62.5	100	76.9	100	100	100
Telomerase pseudoknot, human	47	1	100	100	100	100	100	100
tRNA(asp), yeast	75	0	81.0	100	89.5	95.2	95.2	95.2
TPP riboswitch, E. coli	79	0	46.5	47.6	47.1	95.4	87.5	91.3
SARS corona virus pseudoknot	82	1	69.2	86.3	69.2	84.6	88.0	86.3
cyclic-di-GMP riboswitch, V. cholerae	97	0	85.5	81.0	83.2	89.3	86.2	87.7
SAM I riboswitch, T. tengcongenis	118	1	79.5	91.2	84.9	92.3	97.3	94.7
M-Box riboswitch, B. subtilis	154	0	87.5	91.3	89.4	87.5	91.3	89.3
P546 domain, bI3 group I intron	155	0	55.4	57.4	56.4	94.6	96.4	95.5
Lysine riboswitch, T. maritima	174	1	85.7	94.7	90.0	87.3	88.7	88.0
Group I intron, Azoarcus sp.	214	1	52.4	54.1	53.2	92.1	95.1	93.5
Signal recognition particle RNA, human	301	0	70.0	73.7	71.8	55.0	53.9	54.4
Hepatitis C virus IRES domain	336	1	71.2	74.0	72.5	92.3	96.0	94.1
RNase P, B. subtilis	405	1	55.7	59.3	57.4	75.6	79.8	77.7
Group II intron, O. iheyensis	412	1	87.9	95.9	91.7	93.2	97.6	95.3
Group I intron, T. thermophila	425	1	83.2	85.2	84.2	93.9	91.2	92.5
5’ domain of 23S rRNA, E. coli	511	0	84.0	72.5	77.8	92.4	76.4	83.6
5’ domain of 16S rRNA, E. coli	530	0	73.6	69.0	71.2	89.9	80.6	84.9
**Test set**	**Len**	**PK**	**Iter. HFold**			**ShapeKnots**		
			**sen**	**ppv**	**F**	**sen**	**ppv**	**F**
Fluoride riboswitch, P. syringae	66	1	100	100	100	93.7	93.7	93.7
Adenine riboswitch, V. vulnificus	71	0	100	100	100	100	100	100
tRNA(phe), E. coli	76	0	100	100	100	100	84.0	91.3
5S rRNA, E. coli	120	0	91.4	91.4	91.4	85.7	76.9	81.1
5’ domain of 16S rRNA, H. volcanii	473	0	90.3	82.3	86.1	89.6	82.7	86.0
HIV-1 5’ pseudoknot domain	500	1	45.4	50.4	47.7	100	100	100

### Iterative HFold with SimFold’s suboptimal structures

To further investigate which input structures are good to use when *G*_*big*_ is not known, we use the first 50 suboptimal structures produced by SimFold (including the MFE structure). Then for each RNA sequence we run our methods on all 50 suboptimal structures and choose the one with the lowest free energy as the final result for that RNA sequence. With this approach, the bootstrap 95% percentile confidence interval of average F-measure of HFold and Iterative HFold is (61.80%, 80.63%) and (67.70%, 79.57%) respectively for pseudoknotted structures and (77.17%, 82.35%) and (76.27%, 81.46%) respectively for pseudoknot-free structures. The permutation test indicates that the difference between these results and the corresponding results when input structures are hotspots is not significant. We also test the significance of results of HFold with first 50 suboptimal structures versus Iterative HFold with the same input structures and Iterative HFold with hotspots for both pseudoknotted and pseudoknot-free structures. Although the bootstrap 95% percentile confidence intervals for average F-measures seem different, the permutation test indicates that the difference is not significant. Similarly, results of Iterative HFold with the first 50 suboptimal structures are not significantly better or worse than the result of HFold with hotspots as input structures for both pseudoknotted and pseudoknot-free structures.

### Energy model

In this paper we use the HotKnots V2.0 DP09 [36] energy parameters in our implementation of Iterative HFold. To investigate the degree to which the energy model may be causing mis-predictions by HotKnots V2.0 or Iterative HFold, we considered the degree to which the maximum accuracy structures produced by these methods, i.e., the structure with highest F-measure, is better than the minimum free energy structures. Table 6 presents the difference in bootstrap 95% percentile confidence intervals of average F-measure. If we choose the maximum accuracy structure among the 20 output structures predicted by HotKnots for each RNA sequence, the bootstrap 95% percentile confidence intervals of average F-measure of HotKnots will increase to (84.50%, 91.48%) for pseudoknotted structures of the HK-PK data set (vs. (73.6%, 83.35%) when choosing the lowest energy structure) and (88.32%, 91.08%) for pseudoknot-free structures of the HK-PK-free data set (vs. (76.74%, 81.95%) when choosing the lowest energy structure).

**Table 6:** Comparison of bootstrap 95% percentile confidence interval of average F-measure between the minimum energy structures and the maximum accuracy structures of the HK-PK and the HK-PK-free data sets

**Input structures**	**Min energy**	**Max accuracy**	**Permutation test**
Iter. HFold - hotspots PKed	(72.83, 83.37)	(78.56, 87.05)	Not significant
Iter. HFold - hotspots PK-free	(74.93, 80.26)	(87.70, 90.57)	Significant
Iter. HFold - 50 suboptimals PKed	(67.70, 79.57)	(80.41, 88.14)	Significant
Iter. HFold - 50 suboptimals PK-free	(76.27, 81.46)	(90.05, 93.00)	Significant
HotKnots PKed	(73.60, 83.35)	(84.50, 91.48)	Significant
HotKnots PK-free	(76.74, 81.95)	(88.32, 91.08)	Significant

Similarly, if we compare the maximum accuracy structure output by Iterative HFold with the minimum free energy structure, whether given HotKnots hotspots or the first 50 suboptimal structures to Iterative HFold as input, the bootstrap 95% percentile confidence intervals of average F-measure also show improvement - see Table 6. The difference in improvements is significant in all but one case, namely Iterative HFold on hotspots structures as input for pseudoknotted structures of the HK-PK data set. We conclude that improvements on the energy parameter values for pseudoknotted structures may further improve accuracy of both HotKnots and Iterative HFold.

## Conclusions

In this work we present Iterative HFold, a fast and robust iterative algorithm that matches the accuracy of the best existing pseudoknot prediction methods. Iterative HFold is significantly more accurate than IPknot while matching the accuracy of HotKnots on the HK-PK data set. Iterative HFold is superior to both IPknot and HotKnots on the IP-pk168 data set. Moreover both Iterative HFold and IPknot use less memory and run much faster than HotKnots on long sequences.

Iterative HFold also has lower rate of accuracy deterioration than HFold with loss of information about the true pseudoknot-free structure, so it is more robust than HFold. This is particularly helpful when the given input structure may be unreliable and/or limited information about the true pseudoknot-free structure is available. Iterative HFold is also more accurate than ShapeKnots [47] on the test set of Hajdin et al. [47].

In this work, we compared two different ways to generate pseudoknot-free input structures for input to Iterative HFold, namely the first 50 suboptimal structures produced by SimFold, and HotKnots hotspots. On the HK-PK and HK-PK-free data sets, accuracy of Iterative HFold is not significantly different on each of these. An alternative approach that may be worth exploring in future work would be to use the most highly probable base pairs, as calculated using the partition function [43]. Even better may be to calculate base pair probabilities for base pairs of pseudoknotted RNA structures; however this requires *Θ*(*n*^5^) time. Since HFold finds minimum free energy structure in *O*(*n*^3^) time, conditional on the given input structure, we are currently investigating ways to develop an *O*(*n*^3^)-time partition function version of HFold that can produce pseudoknotted base pair probabilities that are conditional on the given input structure.

Comparing accuracy of the minimum free energy structures with the maximum accuracy structures in this work, we found that, on average, the minimum free energy structure has significantly poorer F-measure than the maximum accuracy structure. This suggests that an improved energy model for pseudoknotted structure prediction may improve accuracy of prediction algorithms for pseudoknotted structures.

Another direction for future work can be to use Iterative HFold for structure prediction of two interacting RNA molecules. Iterative HFold may be well suited for this purpose because, given input structures for each individual input molecule, it allows for modification of these input structures as it explores potential base pairing interactions between the two molecules.

## Data and software availability

Iterative HFold and all data used in this work are freely available at http://www.cs.ubc.ca/~hjabbari/software.php.

## Competing interests

The authors declare that they have no competing interests.

## Authors’ contributions

HJ designed and implemented the algorithms, acquired, analyzed and interpreted the data and drafted the manuscript. AC supervised the research, participated in analysis of data and in revising the manuscript. Both authors read and approved the final manuscript.

## Supplementary Material

Additional file 1 **Pseudocode.** We provide pseudocode of our Iterative HFold algorithm in this section. Click here for file

Additional file 2 **IPknot Performance.** Table 1 provides the bootstrap 95% confidence intervals for average F-measure of IPknot on different data sets and different weight parameters. The energy model in all these experiments is set to McCaskill and level is set to 2 (both default values). Click here for file

Additional file 3 **Robustness Comparison and Correlation to False Positives.** Tables 1 and 2 provide complete presenting robustness comparison of HFold-PKonly, HFold, and Iterative HFold, when provided with different percentage of *G*_*big*_ information of HK-PK and HK-PK-free data
sets. Note that the reported interval in each case is the bootstrap 95% confidence interval for F-measure of the 100 structures with 1≤*α*≤99 percent information about the *G*_*big*_ structure. The 100% information is the bootstrap 95% confidence interval for F-measure when input structure is *G*_*big*_. Table 3 provides Pearson correlation coefficient of HFold and Iterative HFold with HotKnots hotspots, SimFold MFE and SimFold first 50 suboptimal structures. Here FP represents false positive rate and F represents the F-measure as the accuracy measure. Click here for file

Additional file 4 **Time and Memory Comparison.** Tables 1 and 2 provide complete data presenting running time comparison of HFold, Iterative HFold, HotKnots V2.0 and IPknot on the HK-PK data set. Timing is presented in seconds. Tables 3 and 4 provide complete data presenting memory (total heap usage) comparison of HFold, Iterative HFold, HotKnots V2.0 and IPknot on the HK-PK data set. Memory usage is presented in Mega Bytes. Click here for file
