# Supplementary material for: A fast and robust iterative algorithm for prediction of RNA pseudoknotted secondary structures
Source: BMC Bioinformatics. 2014 May 18;15:147. doi: 10.1186/1471-2105-15-147 (PMC4064103; doi:10.1186/1471-2105-15-147)
Supplement: Additional file 1 — Pseudocode. We provide pseudocode of our Iterative HFold algorithm in this section. [file 1471-2105-15-147-S1.pdf]

ADDITIONAL FILE 1 — ITERATIVE HFOLD PSEUDOCODE

Pseudocode

Following is the pseudocode of Iterative HFold's algorithm.

Algorithm 0.1: Iterative HFold (Sequence S, Structure G)

---

comment: Run methods 1-4 and choose the lowest energy structure

$(G_1, E1) \leftarrow \text{method1}(S, G)$
$G_{final} \leftarrow G_1$
$E_{final} \leftarrow E1$
$(G_2, E2) \leftarrow \text{method2}(S, G)$
if $(E2 < E_{final})$
  then $\begin{cases} G_{final} \leftarrow G_2 \\ E_{final} \leftarrow E2 \end{cases}$
$(G_3, E3) \leftarrow \text{method3}(S, G)$
if $(E3 < E_{final})$
  then $\begin{cases} G_{final} \leftarrow G_3 \\ E_{final} \leftarrow E3 \end{cases}$
$(G_4, E4) \leftarrow \text{method1}(S, G)$
if $(E4 < E_{final})$
  then $\begin{cases} G_{final} \leftarrow G_4 \\ E_{final} \leftarrow E4 \end{cases}$
return (G_{final}, E_{final})

Algorithm 0.2: method1(Sequence S, Structure G)

---

comment: Run HFold and return results
return $(HFold(S, G))$

Algorithm 0.3: METHOD2(Sequence S, Structure G)

$(G_1, E1) \leftarrow$ HFold-PKonly(S, G)
if (G_1 is empty structure)
  then return (G)

  else $\begin{cases} G' \leftarrow (G_1 - G) \\ \textbf{return}\ (\text{method1}(S, G')) \end{cases}$

Algorithm 0.4: METHOD3(Sequence S, Structure G)

procedure OBTAINRELAXEDSTEMS$(G1, G2)$
 $G_{result} \leftarrow G1$
 for each $(i.j \in G2)$
  do
  if $i.j \notin G1$
   then
    comment: extend stems of G1
    if $((i-1).(j+1)$ **or** $(i+1).(j-1)) \in G1$
     then $G_{result} \leftarrow G_{result} \cup i.j$
    comment: include bulges of size 1

     else if $((i-2).(j+1)$ **or** $(i-1).(j+2)$ **or** $(i+1).(j-2)$ **or** $(i+2).(j-1)) \in G1$
     then $G_{result} \leftarrow G_{result} \cup i.j$
    comment: include loops of size 1×1

     else if $((i-2).(j+2)$ **or** $(i+2).(j-2)) \in G1$
     then $G_{result} \leftarrow G_{result} \cup i.j$
    comment: include loops of size 1×2 or 2×1

     else if $((i-3).(j+2)$ **or** $(i-2).(j+3)$ **or** $(i+2).(j-3)$ **or** $(i+3).(j-2)) \in G1$
     then $G_{result} \leftarrow G_{result} \cup i.j$
 return (G_{result})

main
 $G' \leftarrow$ SimFold(S, G)
 $G_{updated} \leftarrow$ OBTAINRELAXEDSTEMS(G, G')
 return $(\text{method2}(S, G_{updated}))$

Algorithm 0.5: METHOD4(Sequence S, Structure G)

$k \leftarrow 1$
$G_{updated} \leftarrow G$
for each disjoint substructure G_k in G
do
$$\begin{cases} S_k \leftarrow \text{subsequence of } S \text{ corresponding to } G_k \\ G'_k \leftarrow \text{SimFold}(S_k, G_k) \\ G'_{k,updated} \leftarrow \textsc{ObtainRelaxedStems}(G_k, G'_k) \\ G_{updated} \leftarrow G_{updated} \cup G'_{k,updated} \end{cases}$$
return (method2($S, G_{updated}$))
