# Supplementary material for: A fast and robust iterative algorithm for prediction of RNA pseudoknotted secondary structures
Source: BMC Bioinformatics. 2014 May 18;15:147. doi: 10.1186/1471-2105-15-147 (PMC4064103; doi:10.1186/1471-2105-15-147)
Supplement: Additional file 2 — IPknot Performance. Table 1 provides the bootstrap 95% confidence intervals for average F-measure of IPknot on different data sets and different weight parameters. The energy model in all these experiments is set to McCaskill and level is set to 2 (both default values). [file 1471-2105-15-147-S2.pdf]

ADDITIONAL FILE 2 — IPKNOT DETAILED PERFORMANCE

IPknot Performance

Table 1. Comparison of bootstrap 95% percentile confidence interval of average F-measure of IPknot with different default settings.

input data set	no refinement $\gamma_1 = 1, \gamma_2 = 1$	no refinement $\gamma_1 = 2, \gamma_2 = 4$	no refinement $\gamma_1 = 2, \gamma_2 = 16$	no refinement $\gamma_1 = 4, \gamma_2 = 8$	refinement (1 iteration) $\gamma_1 = 1, \gamma_2 = 1$	refinement (1 iteration) $\gamma_1 = 2, \gamma_2 = 4$	refinement (1 iteration) $\gamma_1 = 2, \gamma_2 = 16$	refinement (1 iteration) $\gamma_1 = 4, \gamma_2 = 8$
HK - PKed	(40.70, 54.82)	(53.27, 65.91)	(57.31, 69.41)	(54.67, 66.07)	(50.63, 66.80)	(57.88, 71.53)	(59.06, 72.02)	(56.52, 69.26)
HK - PK free	(38.04, 40.42)	(39.35, 41.54)	(38.88, 40.97)	(77.31, 81.72)	(32.52, 36.34)	(37.42, 39.95)	(36.19, 38.60)	(37.18, 39.49)
pk168	(47.00, 56.66)	(56.86, 64.81)	(60.73, 68.87)	(58.17, 66.16)	(58.53, 68.93)	(66.31, 75.39)	(65.98, 75.00)	(64.10, 73.20)
DK-pk16	(64.90, 74.50)	(66.22, 77.80)	(66.70, 79.15)	(65.44, 75.74)	(51.57, 79.59)	(55.99, 78.76)	(55.22, 77.84)	(62.16, 76.88)
