# Supplementary material for: A fast and robust iterative algorithm for prediction of RNA pseudoknotted secondary structures
Source: BMC Bioinformatics. 2014 May 18;15:147. doi: 10.1186/1471-2105-15-147 (PMC4064103; doi:10.1186/1471-2105-15-147)
Supplement: Additional file 3 — Robustness Comparison and Correlation to False Positives. Tables 1 and 2 provide complete presenting robustness comparison of HFold-PKonly, HFold, and Iterative HFold, when provided with different percentage of Gbig information of HK-PK and HK-PK-free data sets. Note that the reported interval in each case is the bootstrap 95% confidence interval for F-measure of the 100 structures with 1≤α≤99 percent information about the Gbig structure. The 100% information is the bootstrap 95% confidence interval for F-measure when input structure is Gbig. Table 3 provides Pearson correlation coefficient of HFold and Iterative HFold with HotKnots hotspots, SimFold MFE and SimFold first 50 suboptimal structures. Here FP represents false positive rate and F represents the F-measure as the accuracy measure. [file 1471-2105-15-147-S3.pdf]

ADDITIONAL FILE 3 — ROBUSTNESS COMPARISON AND CORRELATION TO FALSE POSITIVES.

Comparison of robustness with respect to partial information about input structure.

Tables 1 and 2 provide complete presenting robustness comparison of HFold-PKonly, HFold, and Iterative HFold, when provided with different percentage of G_{big} information of HK-PK and HK-PK-free data sets. Note that the reported interval in each case is the bootstrap 95% confidence interval for F-measure of the 100 structures with $1 \leq \alpha \leq 99$ percent information about the G_{big} structure. The 100% information is the bootstrap 95% confidence interval for F-measure when input structure is G_{big}.

Comparison of Pearson correlation coefficient between false positive rates in input structure and output structures.

Table 3 provides Pearson correlation coefficient of HFold and Iterative HFold with Hot-Knots hotspots, SimFold MFE and SimFold first 50 suboptimal structures. Here FP represents false positive rate and F represents the F-measure as accuracy measure. We used R statistical tools to calculate the correlation coefficients. We used all 20 hotspots and all 50 suboptimals for each RNA sequence in our HK-PK data set for this calculation.

As seen in Table 3, there is a negative correlation between false positive rates and prediction accuracy in every case except for hotspots false positive rate in Iterative HFold, in which case the correlation is close to 0, indicating that there is little correlation. Not surprisingly, the negative correlation is quite strong for the MFE structures, because there are many more false positives in these structures.

Table 1. Comparison of robustness of different versions of HFold on HK-PK and HK-PK-free data sets.

% G_{big}	info.	PKonly	HFold	Iterative
HK-PK	1%	(2.35%, 3.04%)	(47.58%, 59.19%)	(47.95%, 59.62%)
	5%	(11.09%, 13.42%)	(52.50%, 62.24%)	(54.51%, 64.56%)
	10%	(20.41%, 24.43%)	(56.79%, 65.01%)	(60.64%, 69.68%)
	15%	(27.97%, 32.95%)	(60.18%, 67.36%)	(65.08%, 73.36%)
	20%	(35.19%, 40.57%)	(62.77%, 69.19%)	(68.51%, 76.54%)
	25%	(41.07%, 46.93%)	(64.50%, 70.36%)	(70.77%, 78.79%)
	30%	(46.53%, 52.78%)	(65.87%, 71.54%)	(72.54%, 80.85%)
	35%	(51.60%, 57.78%)	(67.65%, 72.96%)	(74.18%, 82.30%)
	40%	(55.68%, 61.87%)	(68.72%, 73.76%)	(75.30%, 83.27%)
	45%	(59.68%, 65.82%)	(69.39%, 74.51%)	(75.85%, 83.74%)
	50%	(63.32%, 69.33%)	(70.25%, 75.17%)	(76.50%, 84.64%)
	55%	(66.71%, 72.65%)	(71.18%, 75.96%)	(77.06%, 85.12%)
	60%	(69.81%, 75.65%)	(72.04%, 76.75%)	(77.71%, 85.53%)
	65%	(72.73%, 78.66%)	(73.10%, 77.78%)	(78.03%, 85.93%)
	70%	(75.28%, 81.25%)	(74.21%, 78.89%)	(78.12%, 86.06%)
	75%	(78.07%, 83.87%)	(75.76%, 80.43%)	(78.41%, 86.34%)
	80%	(80.57%, 86.39%)	(77.19%, 81.82%)	(78.60%, 86.54%)
	85%	(82.73%, 88.52%)	(78.63%, 83.41%)	(78.70%, 86.68%)
	90%	(84.91%, 90.63%)	(80.62%, 85.73%)	(79.01%, 86.97%)
	95%	(87.19%, 92.89%)	(83.18%, 88.73%)	(79.19%, 87.09%)
	99%	(88.70%, 94.42%)	(85.17%, 91.21%)	(79.31%, 87.34%)
	100%	(89.03%, 94.83%)	(85.74%, 91.87%)	(79.36%, 87.41%)
HK-PK-free	1%	(1.43%, 1.51%)	(79.76%, 84.32%)	(79.14%, 83.84%)
	5%	(7.01%, 7.21%)	(83.26%, 86.76%)	(80.98%, 85.14%)
	10%	(14.08%, 14.40%)	(86.13%, 88.99%)	(82.86%, 86.43%)
	15%	(21.12%, 21.56%)	(88.37%, 90.78%)	(84.59%, 87.91%)
	20%	(28.02%, 28.60%)	(89.97%, 92.01%)	(85.67%, 88.86%)
	25%	(34.52%, 35.17%)	(91.10%, 92.97%)	(86.80%, 89.73%)
	30%	(40.70%, 41.44%)	(91.98%, 93.68%)	(87.63%, 90.45%)
	35%	(46.70%, 47.46%)	(92.78%, 94.34%)	(88.52%, 91.20%)
	40%	(52.20%, 52.98%)	(93.34%, 94.85%)	(89.01%, 91.71%)
	45%	(57.39%, 58.19%)	(93.82%, 95.18%)	(89.63%, 92.22%)
	50%	(62.37%, 63.16%)	(94.31%, 95.62%)	(89.99%, 92.58%)
	55%	(66.87%, 67.66%)	(94.59%, 95.87%)	(90.32%, 92.83%)
	60%	(71.29%, 72.05%)	(94.90%, 96.17%)	(90.77%, 93.26%)
	65%	(75.45%, 76.17%)	(95.17%, 96.37%)	(91.08%, 93.54%)
	70%	(79.28%, 80.00%)	(95.40%, 96.59%)	(91.44%, 93.85%)
	75%	(83.01%, 83.71%)	(95.57%, 96.74%)	(91.76%, 94.16%)
	80%	(86.56%, 87.21%)	(95.75%, 96.88%)	(92.02%, 94.37%)
	85%	(89.86%, 90.50%)	(95.87%, 97.00%)	(92.60%, 94.82%)
	90%	(93.11%, 93.70%)	(95.98%, 97.12%)	(92.99%, 95.22%)
	95%	(96.08%, 96.68%)	(96.06%, 97.19%)	(93.46%, 95.64%)
	99%	(98.41%, 98.99%)	(96.09%, 97.23%)	(93.76%, 95.98%)
	100%	(98.96%, 99.54%)	(96.11%, 97.24%)	(93.85%, 96.07%)

TABLE 2. Comparison of robustness of different versions of HFold over all structures of our HK-PK and HK-PK-free data sets.

% G_{big} info.	PKonly	HFold	Iterative
1%	(1.63%, 1.81%)	(73.76%, 78.56%)	(73.33%, 78.22%)
5%	(7.87%, 8.50%)	(77.22%, 81.32%)	(76.05%, 80.32%)
10%	(15.41%, 16.48%)	(80.33%, 83.80%)	(78.75%, 82.51%)
15%	(22.61%, 23.89%)	(82.72%, 85.81%)	(81.05%, 84.44%)
20%	(29.61%, 31.03%)	(84.41%, 87.26%)	(82.64%, 85.85%)
25%	(36.01%, 37.50%)	(85.58%, 88.33%)	(83.97%, 87.01%)
30%	(42.07%, 43.64%)	(86.56%, 89.11%)	(85.06%, 88.03%)
35%	(47.90%, 49.46%)	(87.52%, 89.98%)	(86.09%, 88.89%)
40%	(53.14%, 54.65%)	(88.18%, 90.53%)	(86.65%, 89.51%)
45%	(58.12%, 59.57%)	(88.64%, 90.97%)	(87.29%, 90.04%)
50%	(62.81%, 64.23%)	(89.25%, 91.48%)	(87.68%, 90.48%)
55%	(67.10%, 68.45%)	(89.67%, 91.85%)	(88.07%, 90.80%)
60%	(71.23%, 72.59%)	(90.08%, 92.24%)	(88.54%, 91.25%)
65%	(75.11%, 76.48%)	(90.54%, 92.58%)	(88.86%, 91.53%)
70%	(78.72%, 80.05%)	(90.97%, 92.98%)	(89.17%, 91.81%)
75%	(82.19%, 83.56%)	(91.48%, 93.39%)	(89.43%, 92.11%)
80%	(85.51%, 86.85%)	(91.91%, 93.75%)	(89.70%, 92.33%)
85%	(88.58%, 89.93%)	(92.35%, 94.14%)	(90.15%, 92.72%)
90%	(91.57%, 92.92%)	(92.94%, 94.65%)	(90.58%, 93.10%)
95%	(94.38%, 95.75%)	(93.60%, 95.28%)	(90.93%, 93.49%)
99%	(96.54%, 97.91%)	(94.09%, 95.75%)	(91.25%, 93.80%)
100%	(97.03%, 98.44%)	(94.22%, 95.91%)	(91.31%, 93.90%)

TABLE 3. Comparison of correlation between false positive rates in input structure and output structures.

	Iter. HFold F	HFold F
Hotspots - FP	0.04	−0.16
MFE - FP	−0.78	−0.79
Sub50 - FP	−0.35	−0.36
