# Supplementary material for: A fast and robust iterative algorithm for prediction of RNA pseudoknotted secondary structures
Source: BMC Bioinformatics. 2014 May 18;15:147. doi: 10.1186/1471-2105-15-147 (PMC4064103; doi:10.1186/1471-2105-15-147)
Supplement: Additional file 4 — Time and Memory Comparison. Tables 1 and 2 provide complete data presenting running time comparison of HFold, Iterative HFold, HotKnots V2.0 and IPknot on the HK-PK data set. Timing is presented in seconds. Tables 3 and 4 provide complete data presenting memory (total heap usage) comparison of HFold, Iterative HFold, HotKnots V2.0 and IPknot on the HK-PK data set. Memory usage is presented in Mega Bytes. [file 1471-2105-15-147-S4.pdf]

ADDITIONAL FILE 4 — TIME AND MEMORY COMPARISON

Time Comparison

Tables 1 and 2 provide complete data presenting running time comparison of HFold, Iterative HFold, HotKnots V2.0 and IPknot on HK-PK data set. The tables also show required time to get Hotspots as input structure to HFold and Iterative HFold. Timing is presented in seconds.

Memory Comparison

Tables 3 and 4 provide complete data presenting memory (total heap usage) comparison of HFold, Iterative HFold, HotKnots V2.0 and IPknot on HK-PK data set. Memory usage is presented in Mega Bytes.

Table 1. Running time comparison

Name	Len.	Time (S)				
		HFold	Iter. HFold	HotKnots	Hotspots	IPknot
A.tum.RNaseP	400	1.47	5.81	6041.40	0.41	0.72
tobacco-mosaic-virus	214	0.40	5.57	3404.73	0.23	0.19
telo.human	210	0.63	4.43	181.68	0.22	0.20
TMR-00009	196	0.40	8.27	233.82	0.22	0.16
ASE-00131	195	0.30	1.21	203.12	0.22	0.14
ASE-00360	195	0.33	8.22	1665.94	0.22	0.15
ASE-00429	189	0.29	3.18	567.86	0.22	0.13
CRW-00659	170	0.27	4.68	72.71	0.21	0.12
CRW-00641	168	0.24	5.26	53.05	0.21	0.12
CRW-00611	167	0.24	4.39	532.22	0.21	0.09
CRW-00687	153	0.20	3.83	36.05	0.21	0.09
TMR-00047	130	0.27	2.05	31.39	0.20	0.07
Coxsackie	114	0.15	0.64	7.84	0.20	0.05
TMV.R	105	0.15	1.68	19.73	0.20	0.04
HDV-anti	91	0.15	1.19	11.50	0.20	0.04
RFA-00632	91	0.15	0.81	10.74	0.20	0.03
RFA-00636	90	0.15	0.81	4.03	0.20	0.03
HDV	87	0.14	0.79	4.98	0.20	0.03
TYMV	86	0.14	1.20	9.95	0.20	0.03
TMV.L	84	0.15	1.10	10.53	0.19	0.03
CSFV-IRES	76	0.14	1.08	2.64	0.20	0.02
BVDV-IRES	73	0.13	1.07	1.42	0.19	0.02
CoxB3	73	0.13	1.03	1.22	0.19	0.02
satRPV	73	0.13	1.03	6.67	0.19	0.02
PDB-01009	71	0.13	0.56	2.43	0.19	0.03
SARS-CoV	69	0.13	1.01	1.93	0.20	0.02
PDB-01021	68	0.13	0.56	1.02	0.20	0.02
PDB-01023	68	0.13	0.55	1.24	0.20	0.02
EC-S15	67	0.14	1.01	2.87	0.20	0.02
PDB-00944	65	0.13	0.55	1.27	0.20	0.02
Tt-LSU-P3P7	65	0.18	0.56	1.88	0.20	0.02
HCV-229E	61	0.13	0.96	1.05	0.19	0.02
PRRSV-16244B	58	0.13	0.97	2.29	0.19	0.02
PRRSV-LV	58	0.13	0.95	1.44	0.19	0.01
HCV-Ires	56	0.12	0.54	1.47	0.19	0.01
Ec-PK4	52	0.12	0.93	0.89	0.20	0.01
AKV-MuLV	50	0.18	1.22	1.54	0.21	0.01
BaEV	50	0.19	0.93	1.37	0.19	0.01
Cas-Br-E-MuLv	50	0.12	0.92	1.47	0.19	0.01
FeLV	50	0.13	0.91	1.57	0.19	0.01
SNV	50	0.13	0.67	1.04	0.19	0.01
GaLV	49	0.13	0.92	1.44	0.19	0.01
Hs-SRP-pkn	47	0.12	0.53	2.80	0.20	0.01

TABLE 2. Running time comparison - Continued

Name	Len.	Time (S)				
		HFold	Iter. HFold	HotKnots	Hotspots	IPknot
Bt-PrP	45	0.13	0.91	1.21	0.19	0.01
HIV-1-RT-2-3a	45	0.12	0.66	0.34	0.19	0.01
Hs-Prp	45	0.13	0.79	0.76	0.20	0.01
minimalIBV	45	0.12	0.91	0.70	0.20	0.01
HIV-1-RT-2-2b	42	0.13	0.90	0.26	0.19	0.01
HIV-1-RT-2-6b	42	0.12	0.90	0.29	0.19	0.01
Ni-VS	42	0.12	0.91	0.47	0.19	0.01
HIV-1-RT-2-5a	41	0.12	0.90	0.42	0.19	0.01
HIV-1-RT-1-8	39	0.12	0.78	0.24	0.19	0.01
HIV-1-RT-2-1b	39	0.12	0.90	0.26	0.19	0.01
SRV-1	38	0.12	0.90	0.86	0.19	0.01
TMV-L	38	0.13	0.78	1.32	0.19	0.01
HIV-1-RT-1-1	37	0.13	0.90	0.33	0.19	0.01
HIV-1-RT-1-17	37	0.12	0.90	0.33	0.19	0.01
HIV-1-RT-1-3a	37	0.12	0.90	0.36	0.20	0.01
HIV-1-RT-1-6	37	0.12	0.90	0.34	0.19	0.01
HIV-1-RT-1-7	37	0.12	0.90	0.28	0.19	0.01
HIV-1-RT-1-9b	37	0.12	0.90	0.39	0.19	0.01
HIV-1-RT-2-10	37	0.12	0.90	0.35	0.19	0.01
HIV-1-RT-2-11	37	0.12	0.90	0.27	0.19	0.01
HIV-1-RT-2-12	37	0.12	0.78	0.27	0.19	0.01
HIV-1-RT-2-4a	37	0.12	0.89	0.40	0.20	0.01
HIV-1-RT-2-7a	36	0.12	0.89	0.30	0.19	0.01
pKA-A	36	0.12	0.65	0.42	0.19	0.01
EIAV	35	0.12	0.89	0.37	0.19	0.01
FIV	35	0.12	0.89	0.30	0.19	0.01
HIVRT32	35	0.12	0.89	0.31	0.20	0.01
HIVRT322	35	0.12	0.89	0.40	0.20	0.01
HIVRT33	35	0.12	0.89	0.32	0.19	0.01
HIV-1-RT-2-9	34	0.12	0.89	0.27	0.19	0.01
MMTV	34	0.12	0.89	0.71	0.19	0.01
MMTV-vpk	34	0.12	0.89	0.34	0.19	0.01
MMTVgag-pro	34	0.12	0.89	0.95	0.19	0.01
T2-gene32	33	0.12	0.65	0.26	0.19	0.01
Ec-PK1	30	0.12	0.89	0.31	0.20	0.01
LP-PK1	30	0.12	0.89	0.24	0.19	0.01
BWYV	28	0.12	0.88	0.31	0.19	0.01
PEMV	28	0.12	0.89	0.25	0.19	0.01
T4-gene32	28	0.12	0.89	0.26	0.19	0.01
BLV	27	0.12	0.52	0.31	0.19	0.01
BYDV-NY-RPV	27	0.12	0.88	0.30	0.19	0.01
CABYV	27	0.12	0.88	0.27	0.19	0.01
BChV	26	0.12	0.88	0.27	0.19	0.01
PLRV-S	26	0.12	0.88	0.29	0.19	0.01
PLRV-W	26	0.12	0.88	0.29	0.19	0.01

Table 3. Memory usage comparison

Name	Len.	Memory (MB)			
		HFold	Iter. HFold	HotKnots	IPknot
A.tum.RNaseP	400	61.33	61.33	93419.00	5.49
tobacco-mosaic-virus	214	53.85	53.85	44241.76	2.10
telo.human	210	53.71	53.71	8355.85	2.05
TMR-00009	196	53.25	53.25	11833.91	1.81
ASE-00131	195	53.21	53.21	12805.66	1.76
ASE-00360	195	53.21	53.21	55708.86	1.94
ASE-00429	189	53.02	53.02	28543.33	1.77
CRW-00659	170	52.42	52.42	4175.79	1.44
CRW-00641	168	52.36	52.36	3503.01	1.44
CRW-00611	167	52.33	52.33	26824.57	1.42
CRW-00687	153	51.91	51.91	3387.13	1.26
TMR-00047	130	51.24	51.24	2730.81	1.01
Coxsackie	114	50.80	50.80	969.34	0.84
TMV.R	105	50.56	50.56	2354.81	0.78
HDV-anti	91	50.20	50.20	1707.54	0.90
RFA-00632	91	50.20	50.20	1528.68	0.78
RFA-00636	90	50.17	50.17	634.52	0.70
HDV	87	50.10	50.10	755.96	0.68
TYMV	86	50.07	50.07	1478.82	0.62
TMV.L	84	50.02	50.02	1455.98	0.57
CSFV-IRES	76	49.82	49.82	457.97	0.52
BVDV-IRES	73	49.75	49.75	252.15	0.50
CoxB3	73	49.75	49.75	245.71	0.48
satRPV	73	49.75	49.75	1141.53	0.49
PDB-01009	71	49.70	49.70	431.24	1.01
SARS-CoV	69	49.66	49.66	357.78	0.47
PDB-01021	68	49.63	49.63	197.48	0.45
PDB-01023	68	49.63	49.63	268.22	0.45
EC-S15	67	49.61	49.61	534.31	0.44
PDB-00944	65	49.56	49.56	261.16	0.44
Tt-LSU-P3P7	65	49.56	49.56	418.68	0.41
HCV-229E	61	49.47	49.47	213.69	0.41
PRRSV-16244B	58	49.40	49.40	462.97	0.39
PRRSV-LV	58	49.40	49.40	322.98	0.39
HCV-Ires	56	49.35	49.35	329.64	0.38
Ec-PK4	52	49.26	49.26	213.75	0.33
AKV-MuLV	50	49.21	49.21	335.54	0.4
BaEV	50	49.21	49.21	285.20	0.36
Cas-Br-E-MuLv	50	49.21	49.21	328.05	0.40
FeLV	50	49.21	49.21	359.76	0.34
SNV	50	49.21	49.21	221.59	0.34
GaLV	49	49.19	49.19	281.26	0.30
Hs-SRP-pkn	47	49.15	49.15	681.41	0.31

Table 4. Memory usage comparison - continued

Name	Len.	Memory (MB)			
		HFold	Iter. HFold	HotKnots	IPknot
Bt-PrP	45	49.10	49.10	303.82	0.59
HIV-1-RT-2-3a	45	49.10	49.10	39.97	0.33
Hs-Prp	45	49.10	49.10	186.98	0.31
minimalIBV	45	49.10	49.10	162.06	0.30
HIV-1-RT-2-2b	42	49.04	49.04	20.13	0.26
HIV-1-RT-2-6b	42	49.04	49.04	29.99	0.27
Ni-VS	42	49.04	49.04	79.84	0.28
HIV-1-RT-2-5a	41	49.01	49.01	66.23	0.27
HIV-1-RT-1-8	39	48.97	48.97	16.75	0.24
HIV-1-RT-2-1b	39	48.97	48.97	23.33	0.26
SRV-1	38	48.95	48.95	220.73	0.34
TMV-L	38	48.95	48.95	368.74	0.25
HIV-1-RT-1-1	37	48.93	48.93	46.26	0.26
HIV-1-RT-1-17	37	48.93	48.93	49.33	0.24
HIV-1-RT-1-3a	37	48.93	48.93	56.45	0.23
HIV-1-RT-1-6	37	48.93	48.93	49.32	0.25
HIV-1-RT-1-7	37	48.93	48.93	29.82	0.24
HIV-1-RT-1-9b	37	48.93	48.93	62.54	0.32
HIV-1-RT-2-10	37	48.93	48.93	49.32	0.29
HIV-1-RT-2-11	37	48.93	48.93	26.47	0.25
HIV-1-RT-2-12	37	48.93	48.93	26.55	0.24
HIV-1-RT-2-4a	37	48.93	48.93	66.74	0.23
HIV-1-RT-2-7a	36	48.90	48.90	36.30	0.25
pKA-A	36	48.90	48.90	76.08	0.32
EIAV	35	48.88	48.88	65.99	0.23
FIV	35	48.88	48.88	37.03	0.21
HIVRT32	35	48.88	48.88	42.99	0.21
HIVRT322	35	48.88	48.88	69.10	0.25
HIVRT33	35	48.88	48.88	43.22	0.21
HIV-1-RT-2-9	34	48.86	48.86	29.66	0.23
MMTV	34	48.86	48.86	176.54	0.23
MMTV-vpk	34	48.86	48.86	52.92	0.25
MMTVgag-pro	34	48.86	48.86	252.39	0.23
T2-gene32	33	48.84	48.84	23.32	0.22
Ec-PK1	30	48.78	48.78	42.94	0.23
LP-PK1	30	48.78	48.78	16.53	0.19
BWYV	28	48.73	48.73	42.48	0.18
PEMV	28	48.73	48.73	20.09	0.18
T4-gene32	28	48.73	48.73	26.47	0.19
BLV	27	48.71	48.71	42.56	0.18
BYDV-NY-RPV	27	48.71	48.71	42.25	0.18
CABYV	27	48.71	48.71	32.66	0.23
BChV	26	48.69	48.69	29.43	0.17
PLRV-S	26	48.69	48.69	36.06	0.18
PLRV-W	26	48.69	48.69	35.89	0.17
